# Relationships Between Leaf Coloration Changes, Cellular Structure, Photosynthetic Physiology, and Hydraulic Traits in *Liquidambar formosana* Hance Under Drought Stress in Autumn

**DOI:** 10.3390/plants15081173

**Published:** 2026-04-10

**Authors:** Mengting Li, Xiongsheng Liu, Renjie Wang, Ying Jiang, Yufei Xiao, Rongyuan Fan, Yong Wang, Jing Huang, Fengfan Chen

**Affiliations:** 1 Guangxi Key Laboratory of Superior Timber Trees Resource Cultivation, Guangxi Forestry Research Institute, Nanning 530002, China; lmt141524@163.com (M.L.); liuxiongsheng1988@163.com (X.L.); wangrj1991@foxmail.com (R.W.); csfujiangying@163.com (Y.J.); xiaoyufei33@163.com (Y.X.); rongyuanfan107@gmail.com (R.F.); 20200100036@csuft.edu.cn (Y.W.); 2 Hunan Academy of Forestry, Changsha 410018, China; gavinhj@163.com

**Keywords:** drought stress, *Liquidambar formosana* Hance, leaf color, cellular structural features, photosynthetic characteristics, leaf hydraulic properties

## Abstract

*Liquidambar formosana* Hance, a tree species in subtropical broad-leaved forests, exhibits a striking autumn leaf coloration. However, how drought stress during this period influences leaf color change remains poorly understood. In this study, two-year-old seedlings were subjected to four drought gradients. Leaf color parameters, pigment contents, cellular structure, photosynthetic physiology, and hydraulic properties were systematically measured throughout the leaf color transition period. The results show that, with increasing drought severity, leaf red-green coordinate a* increased significantly during early-to-middle stress (S1–S3), while lightness L* and yellow-blue coordinate b* increased at late stress (S4). Chlorophyll (Chl) content continuously decreased, anthocyanins (Ant) peaked at mid-stress, and carotenoids (Car) became enriched at late stress. Leaf cellular structure and hydraulic parameters declined, photosynthetic function was inhibited, and antioxidant enzyme activities showed an initial increase followed by a decrease. Correlation analysis and Random Forest models revealed that L* was strongly associated with superoxide dismutase (SOD) activity, carotenoid-to-chlorophyll (Car/Chl) ratio, and net photosynthetic rate (*P_n_*); a* was closely linked to osmotic potential at full saturation (*Ψ*_sat_), relative water content at the turgor loss point (RWCtlp), SOD activity, Car/Chl ratio, anthocyanin-to-chlorophyll (Ant/Chl) ratio, Ant content, transpiration rate (*T_r_*), *P_n_*, and main vein thickness (Mvt), while b* was primarily correlated with *Ψ*_sat_, Car/Chl ratio, SOD activity, Ant/Chl ratio, and *P_n_*. These statistical associations suggest multiple physiological processes are involved in leaf color change. Based on these findings, we propose a hypothetical sequence: drought initially disrupts leaf water status, leading to structural atrophy and hydraulic decline, followed by photosynthetic inhibition, activated antioxidant defense, and altered pigment accumulation, which are correlated with the sequential leaf color transition from green to red to yellow-orange in this species.

## 1. Introduction

Global climate change has led to rising temperatures and more frequent extreme weather events, resulting in intensifying water scarcity and expanding drought areas worldwide [1]. As one of the most prevalent abiotic stresses, drought severely constrains plant growth, development, and natural geographical distribution [2]. Plants exhibit diverse physiological and morphological responses to water deficit, among which leaf color change serves as an intuitive phenotypic indicator of drought stress [3].

Leaf coloration is fundamentally determined by the relative proportions of chlorophyll (Chl), carotenoids (Car), and anthocyanins (Ant) within leaf tissues [4]. Under drought stress, pigment dynamics are tightly coupled with cellular structural integrity, photosynthetic performance, antioxidant defense systems, and hydraulic function [5,6,7]. Previous research has demonstrated that moderate-to-severe drought induces shrinkage of palisade parenchyma cells, collapse of spongy mesophyll tissue, reduced intercellular air spaces, and stomatal closure [8]. These changes collectively inhibit photosynthesis, limit the energy and substrate supply required for pigment biosynthesis, accelerate chlorophyll degradation, and ultimately trigger leaf chlorosis [8,9]. Concurrently, drought promotes excessive accumulation of reactive oxygen species (ROS) and activates key antioxidant enzymes including superoxide dismutase (SOD) and peroxidase (POD), which further modulate pigment metabolism and subsequent leaf coloration [10,11]. However, systematic investigations into how these physiological processes interact in response to drought and correlate with autumn leaf color transition remain largely insufficient.

Although prior studies have examined drought-induced physiological alterations from various angles, most have focused on single-pigment components or isolated regulatory pathways, leaving a comprehensive and integrated research framework unestablished. Notably, targeted research on the autumn leaf color transition stage of deciduous tree species is scarce, and the relationship between leaf color and leaf hydraulic properties under drought conditions remains poorly documented. Leaf hydraulic traits represent critical parameters for evaluating plant water transport efficiency and drought adaptability, directly governing plant productivity and water-use strategies [12]. Leaf color acts as an outward phenotypic reflection of plant water status, while hydraulic traits underpin the core physiological capacity for water transport, water retention, and drought tolerance. These two attributes are intrinsically interconnected via pigment metabolic reprogramming, cellular structural remodeling, and the coupling of photosynthetic and hydraulic processes [13]. Thus, a systematic study integrating cellular structure, photosynthetic physiology, antioxidant systems, and hydraulic traits with drought-induced leaf color variation is essential for advancing our understanding of plant drought response mechanisms.

*Liquidambar formosana* Hance, a deciduous arbor species in the family Altingiaceae, is native to subtropical broad-leaved forests. It is extensively cultivated as an ornamental tree, owing to its stunning autumn foliage, which gradually shifts from green to brilliant red, purple, and orange-yellow hues [14]. To date, studies on leaf coloration in *L. formosana* have predominantly centered on physiological metabolism [14] and molecular regulatory mechanisms underlying leaf color change under natural seasonal senescence [15,16]. In contrast, research exploring how drought stress, particularly during the critical autumn leaf color transition period, modulates its leaf color variation remains limited. Subtropical autumns are typified by declining precipitation and heightened aridity, rendering drought a non-negligible environmental factor shaping the natural leaf color transition of *L. formosana*.

To address these research gaps, the present study utilized two-year-old *L. formosana* seedlings and imposed four drought gradients during the autumn leaf color transition phase. We systematically quantified leaf color parameters, pigment contents, cellular structure, photosynthetic physiology, antioxidant enzyme activities, and hydraulic properties. The objectives were to investigate the impacts of drought stress on leaf coloration, physiological characteristics, and hydraulic performance of *L. formosana*, and to identify the key physiological factors associated with leaf color variation. We proposed two hypotheses: (1) drought stress significantly alters leaf color parameters, pigment contents, photosynthetic physiology, antioxidant enzyme activities, and hydraulic properties of *L. formosana*, with these responses exhibiting consistent dynamic changes as drought severity increases and stress duration prolongs; and (2) leaf hydraulic traits, photosynthetic parameters, and antioxidant enzyme activities are key physiological factors closely correlated with leaf color variation in *L. formosana* under drought stress. This study aims to provide a theoretical basis for the cultivation management and landscape quality regulation of *L. formosana* under the context of ongoing climate change.

## 2. Results

### 2.1. The Effects of Drought Stress on Parameters of Leaf Color and Leaf Morphology

**Figure 1:**
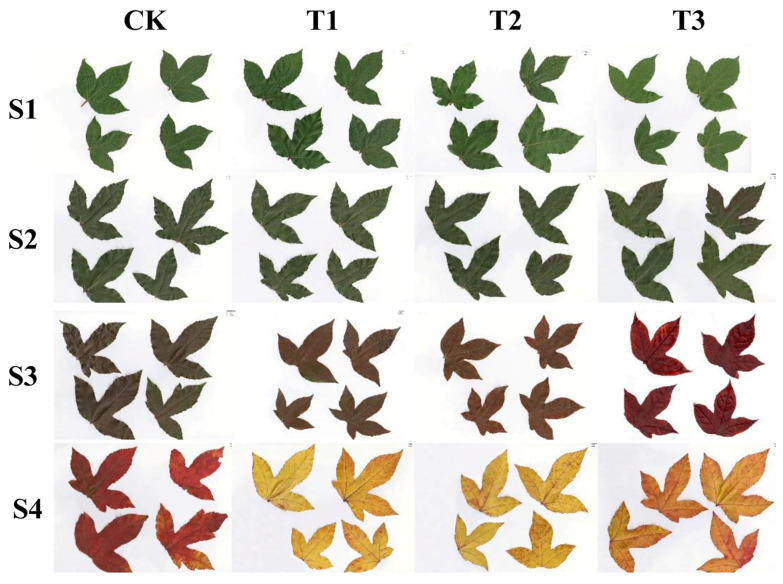
Leaf colors of *Liquidambar formosana* Hance at four periods under different drought treatments. Note: CK, control treatment; T1, mild drought treatment; T2, moderate drought treatment; T3, severe drought treatment; S1, the 1st sampling period (10 November 2021); S2, the 2nd sampling period (30 November 2021); S3, the 3rd sampling period (20 December 2021); S4, the 4th sampling period (9 January 2022).

At the S1 period, the four leaves under drought treatments exhibited a green color (Figure 1); at the S2 period, as the duration of drought stress increased, the leaves under all four treatments appeared dark green; at the S3 period, the leaves under the CK treatment appeared dark green, while those under T1, T2, and T3 treatments exhibited a deep red color, with the red intensity deepening as drought severity increased; by the S4 period, the leaves under CK treatment turned red, those under T1 and T2 treatments appeared yellow, and the leaves under T3 treatment exhibited an orange-red color (Figure 1).

**Figure 2:**
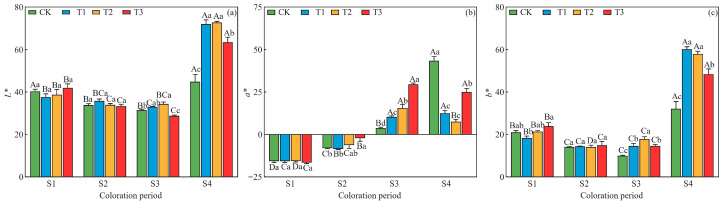
Changes in parameters of leaf color at four periods under different drought treatments. (**a**) Brightness (L*); (**b**) Red-green attribute (a*); (**c**) Yellow-blue attribute (b*) Data are presented as means ± standard error (*n* = 3). Different uppercase letters indicate significant differences among sampling periods, while different lowercase letters indicate significant differences among drought treatments within the same period (*p* < 0.05, according to Duncan’s test for one-way ANOVA).

At the S1 period, there were no significant differences (*p* > 0.05) among parameters of leaf color L*, a*, and b* under different drought treatments (Figure 2); at the S2 period, no significant differences were found in L* and b* among different treatments, while the a* value under T3 treatment was significantly higher than those under the CK, T1, and T2 treatments; at the S3 and S4 periods, both L* and b* exhibited a trend of initial increase followed by decrease in increasing drought severity. The L* value reached its maximum under T2 treatment, while b* values under T1 and T2 treatments were significantly higher than those under other drought treatments (Figure 2); at the S3 period, a* gradually increased with increasing drought severity, with the a* value under T3 treatment (29.6) being markedly higher than that under CK treatment (3.7), which corresponded to the striking visual leaf color shift from dark green to deep red; in contrast, at the S4 period, a* exhibited a trend of an initial decrease followed by an increase with increasing drought severity, with those under the CK treatment exhibiting the highest a* value of 43.4 (Figure 2).

Under the same drought treatment, both L* and b* displayed a trend of initial decrease followed by an increase over time, reaching their maximum values at the S4 period (Figure 2); under CK and T1 drought treatments, a* exhibited a gradual trend of an increase over time; however, under T2 and T3 treatments, a* exhibited a trend of an initial increase followed by a decrease over time, reaching its maximum value at the S3 period (Figure 2).

At the S1 period, there were no significant differences (*p* > 0.05) in the eight indices of leaf morphological structures of leaves among those under different drought treatments (Table 1); at the S2 period, significant differences (*p* < 0.05) were observed in four of the eight indices among different treatments: upper epidermis thickness (Uep), spongy tissue thickness (St), xylem thickness (Xt), and main vein thickness (Mvt); at the S3 period, significant differences were found in Uep, palisade tissue thickness (Pt), St, Xt, and Mvt among different treatments; at the S4 period, significant differences were observed in Uep, lower epidermis thickness (Lep), Pt, St, Xt, and Mvt among different treatments (Table 1). At each period, the indices showing significant differences among drought treatments all exhibited a gradual trend of a decrease in value in response to increasing drought severity (Table 1).

Under the CK treatment, among the eight indices of morphological structure of leaves across different periods, only Lep showed no significant difference, while the other seven indices exhibited significant differences, all displaying a gradual trend of a decrease with prolonged stress duration. Under T1, T2, and T3 treatments, the palisade-to-spongy ratio (PSR) showed no significant difference across different period, while Uep, Lep, Pt, St, Xt, Mvt, and LT exhibited significant differences, all demonstrating a gradual trend of a decrease with prolonged stress duration.

### 2.2. The Effects of Drought Stress on Leaf Pigment Contents, Enzyme Activities, and Photosynthetic Characteristics

**Figure 3:**
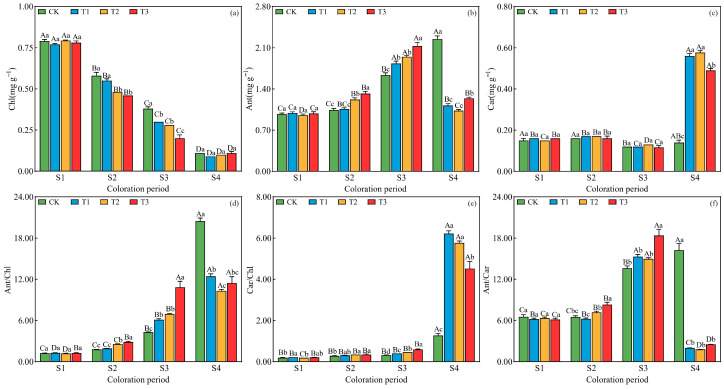
Changes in pigment content in *L. formosana* leaves under different drought treatments at four periods. (**a**), chlorophyll (Chl), (**b**), anthocyanin (Ant), (**c**), carotenoids (Car), (**d**), anthocyanin/chlorophyll (Ant/Chl), (**e**), carotenoids/chlorophyll (Car/Chl) and (**f**), anthocyanin/carotenoids (Ant/Car) in leaves of *L. formosana*. Pigment contents are expressed per gram of fresh weight (mg·g^−1^ FW). Data are presented as means ± standard error (*n* = 3). Different uppercase letters indicate significant differences among sampling periods, while different lowercase letters indicate significant differences among drought treatments within the same period (*p* < 0.05, according to Duncan’s test for one-way ANOVA).

At the S1 period, no significant differences were detected among treatments for any pigment parameter (*p* > 0.05, Figure 3). From the S2 period onward, drought stress systematically altered pigment composition. At S2 and S3, chlorophyll (Chl) content progressively decreased with increasing drought severity, whereas anthocyanin (Ant) content and the Ant/Chl ratio increased markedly under moderate to severe drought (T2 and T3), peaking at S3. Carotenoid (Car) content remained largely unchanged until S4, when it became significantly enriched under T1 and T2 treatments, accompanied by an increased Car/Chl ratio. At the S4 period, distinct pigment responses were also observed for anthocyanins: Ant content and the Ant/Chl ratio exhibited a secondary increase under CK, reaching their highest levels across all treatments, while under drought treatments they continued to decline (Figure 3).

Temporal dynamics within each treatment revealed that Chl content consistently declined with prolonged stress across all treatments. Under control conditions (CK), Ant and Ant/Chl gradually increased over time, reflecting natural autumn senescence. In contrast, under drought treatments (T1–T3), Ant and Ant/Chl exhibited a clear unimodal pattern, rising to a maximum at S3 and declining thereafter. Notably, Car content under T1, T2, and T3 reached its highest level at the final sampling period (S4), coinciding with the yellow-orange leaf coloration observed at this stage (Figure 3).

**Figure 4:**
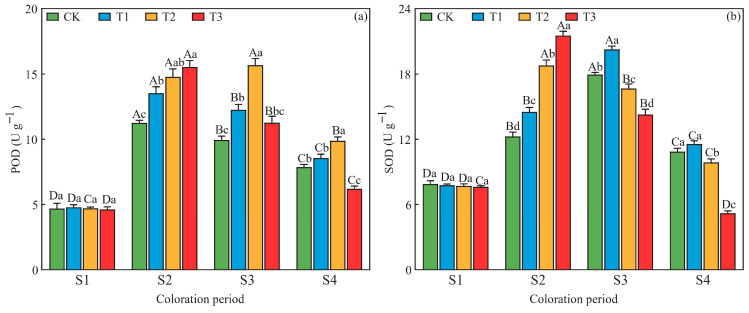
Effects of drought stress on the activities of (**a**) peroxidase (POD) and (**b**) superoxide dismutase (SOD) in *L. formosana* leaves under different drought treatments at four periods. Data are presented as means ± standard error (*n* = 3). Different uppercase letters indicate significant differences among sampling periods, while different lowercase letters indicate significant differences among drought treatments within the same period (*p* < 0.05, according to Duncan’s test for one-way ANOVA).

At S2, S3, and S4, significant differences in POD and SOD activities were observed among drought treatments (Figure 4). At S2, both enzyme activities increased progressively with drought severity, peaking under T3 with values 38.0% (POD) and 75.7% (SOD) higher than CK. At S3 and S4, both enzymes exhibited a unimodal pattern across treatments: POD activity peaked under T2 at both periods, exceeding CK by 57.7% (S3) and 31.3% (S4), whereas SOD activity was highest under T1, with increases of 12.9% (S3) and 6.3% (S4) relative to CK. Temporal dynamics within each treatment also revealed consistent unimodal responses to prolonged stress. POD activity generally peaked at S2 under CK, T1, and T3, but at S3 under T2. SOD activity reached its maximum at S3 under CK and T1, but at S2 under T2 and T3 (Figure 4).

**Figure 5:**
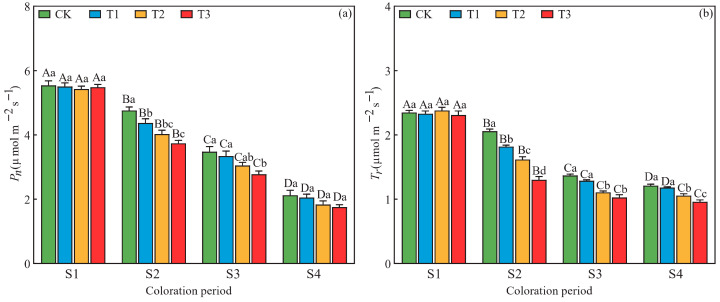
Effects of drought stress on (**a**) net photosynthetic rate (*P_n_*) and (**b**) transpiration rate (*T_r_*) in *L. formosana* leaves under different drought treatments at four periods. Data are presented as means ± standard error (*n* = 3). Different uppercase letters indicate significant differences among sampling periods, while different lowercase letters indicate significant differences among drought treatments within the same period (*p* < 0.05, according to Duncan’s test for one-way ANOVA).

Drought stress exerted significant effects on net photosynthetic rate (*P_n_*) and transpiration rate (*T_r_*) (Figure 5). At the S2 and S3 periods, leaf *P_n_* significantly decreased in response to increasing drought severity; the lowest *P_n_* values were observed under the T3 treatment, showing decreases of 21.4% and 20.1%, respectively, compared to the CK treatment; at the S2, S3, and S4 periods, *T_r_* exhibited a gradual trend of a decrease with increasing drought severity, with the lowest values achieved under T3 treatment, representing decreases of 36.9%, 24.8%, and 20.7%, respectively, compared with the CK treatment. Furthermore, under all four drought stress treatments, both leaf *P_n_* and *T_r_* significantly decreased with prolonged stress duration (Figure 5).

### 2.3. The Effects of Drought Stress on Hydraulic Properties of Leaves

**Figure 6:**
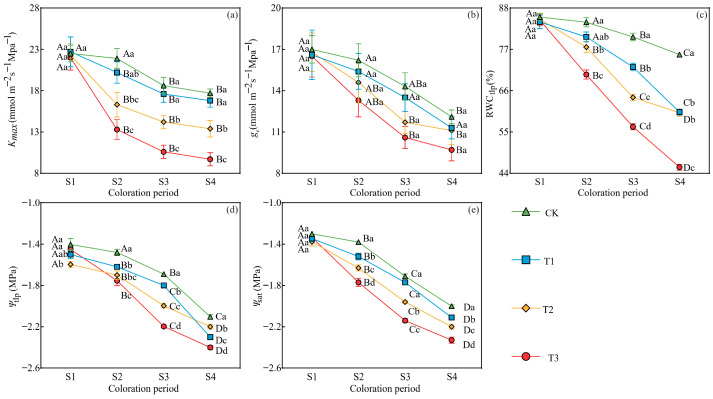
Effects of drought stress on (**a**) maximum leaf hydraulic conductivity (*K_max_*), (**b**) epidermal conductance (*g_e_*), (**c**) relative water content at the turgor loss point (RWC_tlp_), (**d**) water potential at the turgor loss point (*Ψ*_tlp_), and (**e**) osmotic potential at full saturation (*Ψ*_sat_) in *L. formosana* leaves under different drought treatments at four periods. Data are presented as means ± standard error (*n* = 3). Different uppercase letters indicate significant differences among sampling periods, while different lowercase letters indicate significant differences among drought treatments within the same period (*p* < 0.05, according to Duncan’s test for one-way ANOVA).

At the S2, S3, and S4 periods, significant differences (*p* < 0.05) were observed among the different drought treatments in terms of maximum leaf hydraulic conductivity (*K_max_*), relative water content at the turgor loss point (RWC_tlp_), water potential at the turgor loss point (*Ψ*_tlp_), and osmotic potential at full saturation (*Ψ*_sat_). All these parameters exhibited a gradual trend of a decrease with increasing drought severity, with those under the T3 treatment consistently showing the lowest levels (Figure 6). Under four drought treatments, significant differences were noted across different periods in *K_max_*, the epidermal conductance of leaves (*g_e_*), RWC_tlp_, *Ψ*_tlp_, and *Ψ*_sat_, with all these parameters exhibiting a gradual trend of a decrease in response to prolonged stress duration (Figure 6); at the S4 period, compared with the CK treatment, the *K_max_* of leaves under T1, T2, and T3 treatments decreased by 5.1%, 24.3%, and 45.2%, respectively; RWC_tlp_ decreased by 20.2%, 20.5%, and 39.7%, respectively; *Ψ*_tlp_ decreased by 9.5%, 4.8%, and 14.3%, respectively; and *Ψ*_sat_ decreased by 5.5%, 10.0%, and 16.5%, respectively (Figure 6).

### 2.4. Relationships Between Leaf Color and Morphological Structure, Pigment Content, Photosynthetic Physiology, and Hydraulic Properties Under Drought Stress

**Figure 7:**
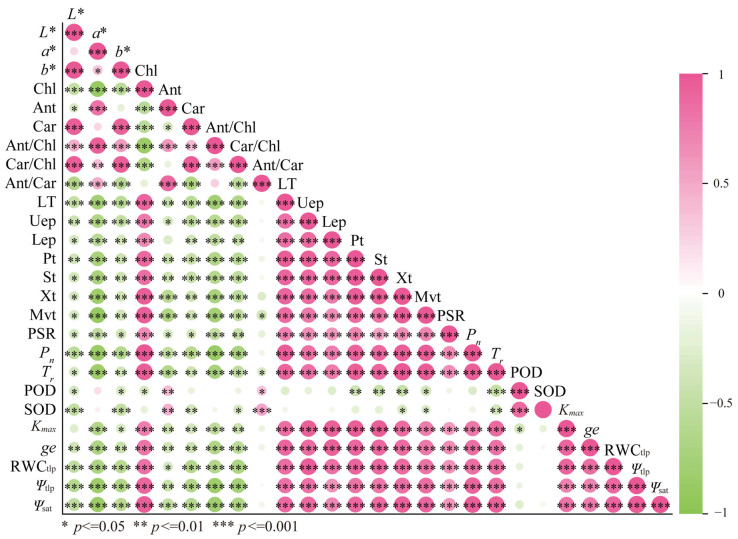
The correlations among leaf color, pigment content, morphological structure, photosynthetic characteristics, enzyme activity, and hydraulic properties of *L. formosana* under drought stress. Note: The circle size represents the absolute value of correlation coefficients among various parameters.

Among the parameters of leaf color, L*, a*, and b* showed significant negative correlations with Chl, while exhibiting significant positive correlations with Ant/Chl and Car/Chl; L* and b* were significantly positively correlated with Car, and a* showed a significant positive correlation with Ant (Figure 7). With the exceptions of the correlations between L* and *K_max_*, and between a* and POD and SOD activities, significant negative correlations were found between leaf color parameters and all other indices of morphological structure, photosynthesis, enzyme activity, and hydraulic properties (Figure 7). Among the indices of pigment concentration, Chl showed significant positive correlations with the indices of morphological structure, photosynthesis, and hydraulic properties, a significant negative correlation with POD, but no significant correlation with SOD; while Ant was significantly positively correlated with indices of enzyme activity, showed no significant correlation with Lep, and was significantly negatively correlated with other indices of morphological structure, photosynthesis, and hydraulic property. Car, Ant/Chl, and Car/Chl were significantly negatively correlated with indices of morphological structure, photosynthesis, and hydraulic property; Car and Car/Chl showed no significant correlation with POD but were significantly negatively correlated with SOD (Figure 7).

**Figure 8:**
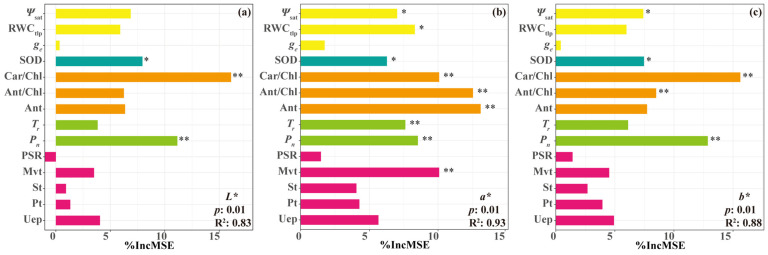
Random Forest analysis of factors influencing leaf color parameters in *L. formosana* under drought stress. (**a**) Predictors of leaf lightness (L*); (**b**) Predictors of red-green attribute (a*); (**c**) Predictors of yellow-blue attribute (b*). In the figure, different colors represent different categories of indicators: pink represents leaf morphological structure, green represents photosynthesis, orange represents pigments, cyan represents enzyme activity, and yellow represents hydraulic properties. Significance levels are indicated as * *p* < 0.05, ** *p* < 0.01.

The Random Forest model further revealed that SOD activity, Car/Chl ratio, and *P_n_* were identified as significant predictors of L* (*p* < 0.05, R^2^ = 0.83); *Ψ*_sat_, RWCtlp, SOD, Car/Chl, Ant/Chl, Ant, *T_r_*, *P_n_*, and Mvt emerged as significant predictors of a* (*p* < 0.05, R^2^ = 0.93); and *Ψ*_sat_, SOD, Car/Chl, Ant/Chl, and *P_n_* were significant predictors of b* (*p* < 0.05, R^2^ = 0.88) (Figure 8). These high R^2^ values indicate strong statistical associations and predictive power of the model, rather than direct regulatory control over leaf color parameters under drought stress.

## 3. Discussion

### 3.1. Relationship Between Leaf Color, Leaf Pigments, and Cellular Structure Under Drought Stress

Pigments serve as the material basis for leaf coloration in colored-leaf plants, and their types, proportions, and distribution directly determine leaf color variation [6]. As the core pigment responsible for leaf greenness, the degradation of chlorophyll (Chl) under drought stress creates conditions for the manifestation of Ant-mediated red hues and Car-mediated yellow hues [17,18], which is consistent with the significant negative correlations observed between Chl and leaf color parameters. In this study, L*, a*, and b* were significantly correlated with pigment content: the carotenoid-to-chlorophyll ratio (Car/Chl) was the key factor associated with L*, while Ant, Ant/Chl, and Car/Chl were the key factors associated with a*, and Ant/Chl and Car/Chl were the key factors associated with b*. These findings indicate that changes in individual pigment components under drought stress cannot fully explain the complex leaf color variations in *L. formosana*; rather, the proportional relationships among pigments play an important role in leaf color variation, consistent with previous findings in *Cotinus coggygria* [2] and *Ziziphus jujuba* Mill. cv. *‘Lingwuchangzao’* [18].

The response of *L. formosana* leaf color to drought stress exhibited distinct temporal patterns, closely linked to dynamic changes in pigment composition. During the early-to-middle-stress periods (S1–S3), leaves gradually reddened, and Ant content and the Ant/Chl ratio increased significantly. As protective pigments against environmental stress, anthocyanins serve dual functions: on the one hand, they alleviate damage to the photosynthetic system by scavenging reactive oxygen species (ROS) and filtering excess light energy [19,20]; on the other hand, their accumulation directly deepens leaf red coloration, consistent with the significant positive correlation between Ant and a* observed in this study and with previous findings that Ant accumulation is the primary cause of reddening in *L. formosana* leaves [7]. At the late stress period (S4), leaf color differed among treatments: Car content increased significantly, and b* values rose markedly, indicating a shift in photoprotective strategy from Ant-mediated pigment screening during early to middle stress to carotenoid-based non-photochemical quenching at the late stage [19,21,22]. This strategic shift represents an adaptive response coordinating photoprotection with carbon metabolic balance under prolonged water limitation, while also directly contributing to the phenotypic transition from red to yellow leaves.

Plant morphological structure underpins physiological function, and internal anatomical features reflect environmental adaptability [23,24,25]. In this study, with increasing drought severity and duration, leaf anatomical parameters (upper epidermis thickness, palisade tissue thickness, spongy tissue thickness, xylem thickness, and main vein thickness) all decreased significantly, indicating overall structural atrophy, consistent with previous findings that drought induces cell contraction and tissue thinning [26]. Changes in leaf anatomy influence color expression by affecting pigment distribution and light reflection and absorption. Reduced leaf thickness may enhance light penetration, indirectly affecting leaf color parameters; reduced palisade tissue thickness is often accompanied by a decrease in chloroplast number; changes in upper epidermis thickness may affect leaf glossiness and thus L* value [27,28]. Random forest analysis identified main vein thickness (Mvt) as a key factor associated with a*, which may be related to its role as a conduit for water and nutrient transport, where changes in thickness affect leaf water status and pigment metabolism [29].

### 3.2. Relationship Between Leaf Color, Photosynthetic Characteristics, and Enzyme Activities Under Drought Stress

Antioxidant enzyme activities and photosynthetic characteristics are core physiological indicators of plant drought tolerance and are closely linked to leaf color parameters and pigment content. Their dynamic patterns reflect the integrated physiological and metabolic adaptation of *L. formosana* to drought stress. Drought stress initially disrupts water balance, leading to significant reductions in net photosynthetic rate (*P_n_*) and transpiration rate (*T_r_*), driven by both stomatal and non-stomatal factors. Stomatal closure reduces stomatal conductance and epidermal conductance (*g_e_*), directly limiting CO_2_ uptake [8,30,31]; non-stomatal factors include reduced Chl content and decreased photochemical efficiency. In this study, both Chl content and *g_e_* declined progressively with increasing drought severity, showing significant positive correlations with *P_n_* and *T_r_*, indicating that both stomatal and non-stomatal factors collectively constrain photosynthesis under drought stress. Reduced *P_n_* not only indicates weakened carbon assimilation, but also triggers decreased light-use efficiency, leading to excess excitation energy and accelerated ROS production [32,33]. Consequently, photosynthetic inhibition may serve as an upstream signal initiating downstream photoprotective responses, thereby driving the accumulation of Ant and Car and the associated leaf color transition.

Under normal conditions, ROS production and scavenging remain balanced; however, drought disrupts this balance, leading to ROS accumulation [34], which causes biomembrane lipid peroxidation and photosynthetic impairment [32,35]. Studies by Almeselmani et al. [36] and Dai et al. [37] demonstrated that antioxidant enzymes can enhance the antioxidant capacity of a plant, the levels of which can determine the strength of free radical scavenging ability, including POD and SOD [32]. SOD acts as the first line of defense by dismutating superoxide anions, while POD is involved in both ROS scavenging and lignin biosynthesis, linking antioxidant defense to structural integrity [38]. During the early stress period (S1–S2), POD and SOD activities increased with drought severity, accompanied by a concurrent rise in Ant content. This combined response represents an integrated enzymatic and non-enzymatic defense network maintaining cellular redox homeostasis [39]. However, at later stress periods (S3–S4), both enzyme activities declined with prolonged stress, exhibiting a unimodal pattern, suggesting that moderate-to-severe stress may exceed the tolerance threshold of the antioxidant system. This is consistent with findings in passion fruit (*Passiflora edulis*) [40].

Random forest analysis (Figure 8) identified SOD activity and *P_n_* as significant predictors of L*, a*, and b* (*p* < 0.05), with *T_r_* additionally associated with a*. These results suggest potential physiological linkages between leaf color parameters and physiological indices: under drought stress, plants mitigate oxidative damage by regulating antioxidant enzyme activity, thereby maintaining pigment metabolism and photosynthetic function, which are associated with leaf color phenotypes. When stress exceeds the plant’s tolerance threshold, enzyme activity declines and photosynthetic function becomes impaired, leading to pigment metabolic disruption and subsequent changes in leaf color parameters.

### 3.3. Relationship Between Leaf Color and Hydraulic Properties Under Drought Stress

The maintenance of homeostasis in the physiological functions of plants relied on the integrity of their hydraulic system, a core functional module governing water absorption, transport, and retention, which determines the adaptability of plants under drought stress [29,41]. In this study, drought stress significantly reduced maximum hydraulic conductivity (*K_max_*), relative water content at the turgor loss point (RWC_tlp_), water potential at the turgor loss point (*Ψ*_tlp_), and osmotic potential at full saturation (*Ψ*_sat_) in *L. formosana* leaves, with these parameters continuously decreasing as stress intensity and duration increased. Such hydraulic responses mainly originated from multiple synergistic effects induced by drought, specifically including the formation of xylem embolism, decreased apoplastic permeability caused by the contraction of mesophyll and vascular bundle sheath cells, and the functional inhibition of aquaporins involved in water transport [42,43,44,45]. Collectively, these responses reflect functional restructuring of the hydraulic system as a key adaptive mechanism to water deficit.

Declining hydraulic function directly constrains water supply to leaves, thereby influencing pigment metabolism and leaf color phenotypes. *K_max_* reflects leaf water transport capacity and is closely linked to maximum photosynthetic rate [46]. In this study, the estimated *K_max_* of *L. formosana* ranged from 9.7 to 22.5 mmol·m^−2^·s^−1^·MPa^−1^, falling within the range reported for angiosperms using direct hydraulic measurements (3.9–36 mmol·m^−2^·s^−1^·MPa^−1^) [47], supporting the reliability of our anatomical estimates for comparative assessment. Reduced xylem hydraulic conductivity limits leaf gas exchange and photosynthetic rate, exacerbating excess light energy and ROS accumulation [29,48]. To counteract the hydraulic dysfunction and the associated excess light energy accumulation, *L. formosana* coordinates physiological adjustments across multiple scales. Studies have shown that leaf hydraulic conductance (*K_leaf_*) strongly influences stomatal behavior and photosynthetic carbon assimilation, with dynamic changes in *K_leaf_* during dehydration contributing to stomatal closure and thus modulating the balance between water loss and photoprotection [49,50]. Furthermore, alterations in xylem hydraulic efficiency can affect leaf water status and nutrient transport, thereby indirectly influencing the biosynthesis and turnover of photosynthetic pigments and flavonoids [45,51]. Collectively, these coordinated adjustments, spanning hydraulic function, stomatal regulation, and antioxidant metabolism, enable *L. formosana* to maintain cellular homeostasis under water deficit. Correlation analysis revealed significant negative correlations between leaf color parameters and hydraulic indices, suggesting that changes in hydraulic status are closely linked to leaf color variation. Random forest analysis further identified *Ψ*_sat_ and RWC_tlp_ as key factors associated with a* and b* values, providing quantitative evidence linking hydraulic properties to leaf color variation.

Based on these correlative findings, we propose a hypothetical “hydraulic–pigment–phenotype” cascade to explain the sequential leaf color transition in *L. formosana* under drought stress. Within this proposed framework, drought initially disrupts hydraulic function, leading to stomatal limitation and reduced photosynthetic rate; photosynthetic limitation exacerbates excess light energy and ROS accumulation, activating antioxidant defense and driving metabolic shifts in protective pigments (Ant and Car); ultimately, temporal changes in pigment composition and ratios are integrated into a staged leaf color phenotype, transitioning from green to red to yellow-orange. We emphasize that this cascade represents a conceptual interpretation derived from observed statistical associations and requires experimental validation in future studies.

Future studies should address the limitations of this work. Experiments across different seasons are needed to isolate drought-specific mechanisms from seasonal senescence, and measurements of compatible osmolytes should be integrated to further elucidate the regulatory network linking drought tolerance and leaf coloration in *L. formosana.*

## 4. Materials and Methods

### 4.1. General Characteristics of the Experimental Site

The drought stress experiment on *L. formosana* was conducted at the Germplasm Resource Nursery of the Guangxi Zhuang Autonomous Region Forestry Research Institute. The nursery, located in the northern suburb of Nanning City, Guangxi Zhuang Autonomous Region, China (N 22°56′, E 108°21′), features a typical southern subtropical monsoon climate characterized by an altitude of 85 m, an annual sunshine duration of approximately 1550 h, average annual temperature of 21.5 °C, average annual rainfall of 1304.2 mm, and average annual relative humidity of 79%.

### 4.2. Experimental Materials

The experimental materials were derived from seeds collected in 2019 from the Red-leaf Forest Park in Debao County, Baise City, Guangxi Zhuang Autonomous Region, China (N 23°21′19″, E 106°39′5″). In September 2021, 240 healthy and uniformly growing two-year-old seedlings (92.3 cm average seedling height, 1.86 cm in average ground diameter) were selected and transplanted into pots containing loam soil. The soil, with an organic matter content of 16.47 g·kg^−1^, pH of 5.3, total nitrogen content of 1.26 g·kg^−1^, total phosphorus of 0.19 g·kg^−1^, total potassium of 42.01 mg·g^−1^, and field capacity (FC, defined as the maximum soil water content that the soil can stably retain) of 30.23%, was used in pots 28 cm in top diameter, 22 cm in bottom diameter, and 31 cm in height. After transplantation, the seedlings were placed in an outdoor rain shelter and watered normally every day.

### 4.3. Experimental Design

Begun on 10 November 2021, the controlled-drought experiment established four drought stress treatments, namely normal watering (CK (control), with soil water content at 100% of field capacity [FC]), mild drought stress (T1, at 75–80% FC), moderate drought stress (T2, at 50–55% FC), and severe drought stress (T3, at 35–40% FC). Each treatment comprised 60 seedlings divided into three replicates with 20 seedlings in each independent replicate. For pigment content and enzyme activity assays, two functional leaves were collected from the same position and orientation from each seedling. Thus, a total of 40 leaves were obtained from each replicate (20 seedlings × 2 leaves). These 40 leaves were pooled and mixed thoroughly to form a single composite sample per replicate, yielding one data point per parameter for each replicate (n = 3). During the experiment, the appropriate FC was maintained in each pot by measuring soil water content daily in the evening, using the weighing method, and replenishing the water loss to keep the soil relative water content within the set range of relative soil water contents. The experiment, lasting 60 days, involved sampling and determination of experimental indices every 20 days for a total of four times, labeled as S1 (10 November 2021, before the start of the experiment), S2 (30 November 2021), S3 (20 December 2021), and S4 (9 January 2022, before leaf fall). During each sampling, leaves from the middle and upper parts of plants within each replicate were collected and mixed to form one composite sample, which, after being brought back to the laboratory and wiped clean, were randomly divided into three portions: one portion for the determination of parameters of leaf color, one portion for the determination of cellular structure, and one portion stored in a −80 °C ultra-low temperature freezer for subsequent determination of pigment content and protective enzyme activity.

### 4.4. Measurements

#### 4.4.1. Determination of Leaf Color

**Figure 9:**
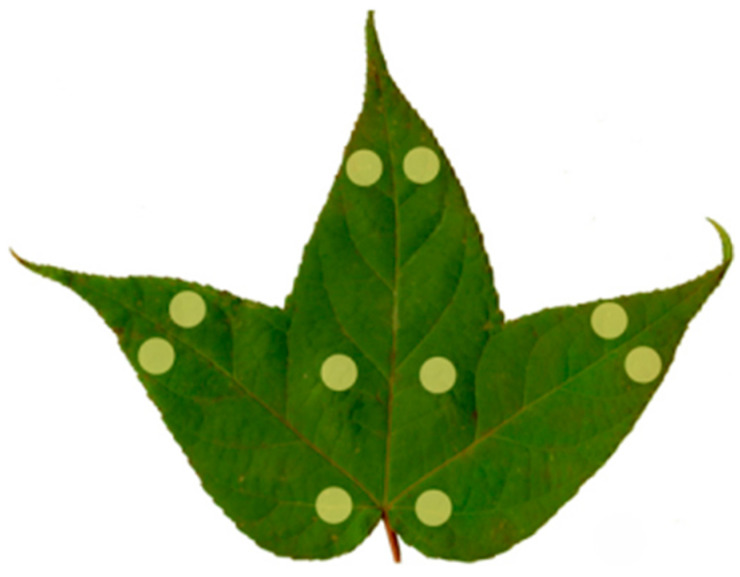
Schematic diagram of the method for determining the leaf colour of *L. formosana*. Yellow circle represents the measuring point of the leaf color parameter.

For each of the four samples, ten randomly selected leaves were used for leaf color determination on the adaxial surface using a spectrophotometer CS-650 (Hangzhou CHNSpec Technology Company Limited, Hangzhou, China) under a D65 light source, at a 10° viewing angle and with an 8 mm aperture, with each leaf determined ten times (Figure 9). The colorimeter directly provided the lightness value L* (the larger the L* value indicates the brighter leaf color), the red-green attribute value a* (the larger the a* value indicates the redder leaf color), and the yellow-blue attribute value b* (the larger the b* value indicates the yellower leaf color) [52].

#### 4.4.2. Determination of Leaf Cell Structural Features

For each sample, five leaves were randomly selected, and, from each leaf, a small section measuring 0.5 cm × 0.5 cm was excised from approximately the upper one-third of each leaf (retaining the main vein) and immediately fixed in FAA fixative (with a volume ratio of ethanol:formaldehyde:glacial acetic acid = 90:5:5) for 24 h. The treated leaves were sectioned using the conventional paraffin sectioning method, with three sections, each with a thickness of 8 μm, prepared from each leaf. After staining with safranin-fast green, the sections were mounted with neutral resin and observed under a Motic BA410 optical microscope (manufactured by Motic (Xiamen) Medical Diagnostic Systems Company Limited, Xiamen, China). For each section, five fields of view were selected from both the leaf veins and mesophyll tissues for observation and photography. Leaf thickness, palisade tissue thickness, spongy tissue thickness, xylem thickness, main vein thickness, and upper and lower epidermis thickness were determined using Digimizer software (version 5.6.0), with ten values determined for each structure [53].

#### 4.4.3. Determination of Leaf Pigment Contents and Protective Enzyme Activities

The content of chlorophylls (Chl) and carotenoids (Car) were determined following the methods described by Lichtenthaler and Wellburn [54]. For each sample, 0.2 g of fresh leaf powder was weighed, mixed with 5 mL of 80% acetone, and placed in a 4 °C refrigerator for 24 h of dark extraction. After filtration of the supernatant, the absorbance values at wavelengths of 445 nm, 645 nm, and 663 nm were determined using a UV-4802 dual-beam spectrophotometer (Unoco (Shanghai) Instrument Company Limited, Shanghai, China), and the contents of Chl and Car were calculated based on the appropriate equations. For anthocyanins (Ant) determination, 1.0 g fresh leaf powder from each sample was weighed, mixed with 10 mL of 1% hydrochloric acid methanol solution, and the mixture was extracted for 5 h at 32 °C. The extract was filtered, diluted five-fold, and the absorbance values at 530 nm and 657 nm were determined using a dual-beam spectrophotometer to calculate the Ant content [55].

Determination of peroxidase (POD) and superoxide dismutase (SOD) activities [56]: 0.1 g of fresh leaf powder from each sample was weighed and placed in a 4 °C precooled mortar to which polyvinylpyrrolidone (PVP), quartz sand and 5 mL of 0.05 mol·L^−1^ phosphate buffer (pH = 7.0) were added. The mixture was ground uniformly for 30 s, poured into a centrifuge tube, and centrifuged at 11,100× *g* for 20 min in a refrigerated centrifuge. The supernatant, serving as the enzyme extract, was employed for the determination of POD and SOD activities.

#### 4.4.4. Determination of Photosynthetic Characteristics and Hydraulic Properties

For each replicate of each treatment, five seedlings were randomly selected. From each seedling, three healthy leaves free from pests and diseases were chosen from the middle and upper part for measurement of photosynthetic and hydraulic parameters. Between 9:00 a.m. and 12:00 p.m., the net photosynthetic rate (*P_n_*) and transpiration rate (*T_r_*) of the leaves were determined using a Li-6400 portable photosynthesis system (manufactured by Beijing Ecotek Technology Company Ltd., Beijing, China). The determination conditions were set as follows: photosynthetic photon flux density (PPFD) at 1000 μmol·m^−2^·s^−2^, CO_2_ concentration at 380 μmol·mol^−1^, leaf chamber temperature at 37 °C under saturated light intensity, and relative humidity at 75% [57].

The maximum leaf hydraulic conductivity (*K_max_*) was identified by employing anatomical methods [12]. At each determination period, for each replicate under each treatment, five seedlings were randomly selected, brought back to the laboratory, and excised underwater by taking leaves, with petioles immersed for over 12 h and transpiration suppressed to allow leaf water absorption to reach saturation. Petioles from three leaves were collected from each seedling to prepare hand-cut sections, stained with 0.5% safranin, and the number and size of xylem vessels were observed under an optical microscope. The hydraulic conductivity of the petiole was calculated using the Hagen-Poiseuille formula, then divided by leaf area to determine leaf hydraulic conductivity. In the experiment, the number of xylem vessels determined for size constituted no less than two-thirds of the total count of vessels in the entire petiole. Vessels, due to their irregular shape, were converted to equivalent circular area diameters to calculate the hydraulic conductivity of a single vessel. The calculation formula is as follows:

(1) $$K_{i}=\frac{{\pi r}^{4}}{8\eta }$$

where *K_i_* represents the hydraulic conductivity of a single vessel, *r* indicates the radius of a circle with the same area as the xylem vessel, and *η* represents the viscosity coefficient of water.

The maximum hydraulic conductivity of leaves (*K_max_*, mmol·m^−2^·s^−1^·MPa^−1^) is as follows:

(2) $$K_{max}=\frac{N}{nA}\sum_{1}^{n}K_{i}$$

where *N* represents the total number of xylem vessels in the petiole, n is the number of vessels selected for determination, and *A* is the leaf area.

Epidermal conductance (*g_e_*) of leaves was determined with reference to the method described by Muchow [58]. For each determination, for each replicate under each treatment, five seedlings were randomly selected. Branches (primary branches from the middle and upper part of each seedling) were cut after sunset, brought back to the laboratory, and excised underwater by taking leaves, which were then immersed in water for over 12 h to achieve saturation. After saturation, the leaves were removed, wiped dry, and quickly weighed for weight at saturation. The leaves were then placed in a dark environment with relatively stable temperature and humidity, and their fresh weight was determined at regular intervals (50 min). The weighing ended when the relative water content of leaves dropped to 60–65% (8 h). After the determination was over, the samples were placed in a 75 °C oven for 48 h and weighed to determine dry weight. The fresh weight of leaves was plotted against water loss time. Then, the data from the linear portion after stomatal closure were selected for linear regression to obtain the epidermal conductance (*g_e_*) of leaves. The calculation equation is as follows:

(3) $$g_{e}=2.31\times 10^{6}\times \frac{∆w}{t}\times \frac{1}{A}\times \frac{1}{∆e}$$

where Δ*w*/*t* is the water loss per unit time (i.e., the slope of the regression line, g·s^−1^); *A* represents leaf area (m^2^); Δ*e* indicates the absolute humidity difference between the leaf and the atmosphere (mm·m^−3^); and 2.31 × 10^6^ represents the unit conversion factor.

Pressure–volume (PV) curves were plotted employing the natural air-drying method proposed by Tyree and Hammel [59]. During each measurement, for each replicate under each treatment, five seedlings were randomly selected. Branches with leaves were cut after sunset, brought back to the laboratory, and excised underwater by taking leaves. After saturation, the leaves were removed, wiped dry, and quickly weighed to obtain the weight at saturation. The leaves were then placed in a pressure chamber to measure their balance pressure. Then, the leaves were removed and allowed to air-dry naturally indoors. Their weight and balance pressure were determined at regular intervals. This process was repeated until the samples reached severe wilting, defined operationally as the point when no stable balance pressure could be obtained upon pressurization. Subsequently, the samples were placed in an oven at 75 °C for 48 h and weighed to determine dry weight. Parameters of the PV curve were calculated by fitting using the PV curve program developed by Schulte and Hinkley [60], specifically osmotic potential at full saturation (*Ψ*_sat_), water potential at the turgor loss point (*Ψ*_tlp_), and relative water content at the turgor loss point (RWC_tlp_).

### 4.5. Statistical Analyses

The data were processed using Microsoft Excel 2021 and one-way analysis of variance (ANOVA) and Duncan’s multiple range test to identify differences between mean values (*p* < 0.05) using R software version 4.4.2. Relationships among parameters of leaf color, morphological leaf structure, pigment concentration, photosynthesis characteristics, enzyme activity, and hydraulic properties were assessed through Pearson’s correlation analysis. To evaluate the relative importance of physiological and structural parameters in explaining leaf color variation, a Random Forest model with a permutation-based significance test was employed. The analysis was performed using the randomForest, rfPermute, and rfutilities packages in R (version 4.4.2). Prior to modeling, backward selection was applied to 23 candidate physiological and structural parameters, resulting in 14 variables retained for final analysis. The Random Forest model was built with 500 decision trees (ntree = 500), and missing values were handled using na.omit. Variable importance was assessed by the percentage increase in mean squared error (%IncMSE) upon random permutation. Statistical significance of variable importance was evaluated using permutation tests (100 permutations) with the rfPermute package; variables with *p* < 0.05 were considered significant predictors of leaf color variation. Overall model significance was tested using the rf.significance function from the rfutilities package with 99 permutations. All graphs and charts were generated using GraphPad Prism 8 software.

## 5. Conclusions

Under drought stress, systematic changes occurred in the parameters of leaf color, pigment composition, antioxidant enzyme activities, photosynthetic characteristics, and hydraulic properties of *L. formosana*. With increasing drought severity and prolonged stress duration, the red-green attribute a* increased significantly at the early to middle periods of stress (S1–S3), while leaf lightness L* and the yellow-blue attribute b* increased significantly at the late period of stress, S4. Chl content continuously decreased with increasing drought stress, Ant accumulated notably at the mid-stress period but decreased later, and Car became relatively enriched at the late period of stress. Meanwhile, both the cellular structural indices and hydraulic property parameters of leaves significantly decreased, photosynthetic function was significantly inhibited, and protective enzyme activities exhibited a trend of an initial increase followed by a decrease in response to increasing stress. Correlation analysis and the Random Forest model revealed that leaf lightness L* was strongly associated with SOD activity, Car/Chl ratio, and net photosynthetic rate (*P_n_*); the red-green attribute a* was closely linked to osmotic potential at saturated water content (*Ψ*sat), relative water content at the turgor loss point (RWCtlp), SOD, Car/Chl, Ant/Chl, Ant content, transpiration rate (*T_r_*), *P_n_*, and main vein thickness (Mvt); while the yellow-blue attribute b* was primarily correlated with *Ψ*_sat_, Car/Chl, SOD, Ant/Chl, and *P_n_*. These statistical associations suggest that multiple physiological processes are involved in leaf color change, although further studies are needed to establish direct regulatory mechanisms. Based on the aforementioned results, we propose the following sequence of events for regulation of the leaf color of *L. formosana* under drought stress. Drought initially disrupts leaf water status, leading to declines in relative water content and water potential. This primary water deficit subsequently induces structural atrophy of leaf tissues and functional decline in the hydraulic system, which in turn triggers photosynthetic inhibition, activates the antioxidant defense system, and drives the metabolic reorganization of protective pigments. The temporal changes in pigment composition and ratios are then integrated into a staged phenotype of leaf color, transitioning from green to red and then to yellow-orange. This study provides a theoretical reference for understanding drought responses in *L. formosana* and offers preliminary insights that may inform future research on cultivation management and landscape applications under changing climatic conditions.

## Figures and Tables

**Table 1:** Leaf morphological structural features at four periods under different drought treatments.

Treatment	Period	Uep (μm)	Lep (μm)	Pt (μm)	St (μm)	Xt (μm)	Mvt (μm)	LT (μm)	PSR
CK	S1	14.5 ± 0.3 Aa	10.6 ± 0.7 Aa	82.1 ± 3.7 Aa	62.7 ± 3.6 Aa	77.1 ± 2.5 Aa	925.3 ± 1.6 Aa	170.5 ± 5.1 Aa	1.31 ± 0.02 Aa
	S2	14.2 ± 0.1 Aa	10.1 ± 0.6 Aa	72.1 ± 1.1 Ba	57.1 ± 0.8 ABa	67.1 ± 1.3 Ba	862.5 ± 0.7 Ba	155.3 ± 2.0 Ba	1.26 ± 0.00 Ba
	S3	13.5 ± 0.1 Ba	10.2 ± 0.8 Aa	66.8 ± 1.8 BCa	54.8 ± 2.4 BCa	52.4 ± 1.4 Ca	723.6 ± 7.6 Ca	147.5 ± 2.6 Ba	1.22 ± 0.02 Ba
	S4	13.1 ± 0.2 Ba	8.9 ± 0.5 Aa	61.5 ± 0.9 Ca	49.2 ± 0.2 Ca	50.5 ± 0.3 Ca	716.3 ± 8.4 Ca	131.3 ± 2.1 Ca	1.25 ± 0.01 Ba
T1	S1	14.2 ± 0.1 Aa	10.2 ± 0.8 Aa	82.3 ± 1.3 Aa	63.3 ± 2.3 Aa	77.2 ± 2.3 Aa	916.3 ± 5.6 Aa	168.1 ± 2.8 Aa	1.30 ± 0.03 Aa
	S2	13.8 ± 0.3 Aab	9.6 ± 0.6 Aa	70.5 ± 2.4 Ba	55.2 ± 1.4 Ba	65.2 ± 1.8 Ba	855.2 ± 4.5 Ba	154.1 ± 5.7 ABa	1.28 ± 0.01 Aa
	S3	12.8 ± 0.4 Ba	9.0 ± 0.5 Aa	64.2 ± 3.4 BCab	53.2 ± 1.2 BCa	50.2 ± 0.8 Ca	714.6 ± 3.3 Cab	143.8 ± 4.4 Ba	1.21 ± 0.04 Aa
	S4	12.2 ± 0.2 Bb	8.8 ± 0.4 Aab	58.2 ± 2.6 Ca	48.6 ± 0.9 Ca	48.9 ± 0.5 Cb	714.2 ± 3.6 Ca	128.5 ± 4.6 Ca	1.20 ± 0.03 Aa
T2	S1	14.1 ± 0.4 Aa	10.6 ± 0.6 Aa	82.5 ± 2.7 Aa	62.6 ± 2.8 Aa	76.4 ± 2.2 Aa	922.2 ± 5.1 Aa	171.6 ± 3.1 Aa	1.32 ± 0.02 Aa
	S2	13.1 ± 0.3 Bbc	8.8 ± 0.5 ABa	63.2 ± 3.3 Bab	48.6 ± 0.9 Bb	58.3 ± 1.1 Bb	836.8 ± 4.3 Bb	150.6 ± 2.7 Ba	1.30 ± 0.04 Aa
	S3	11.3 ± 0.2 Cb	8.1 ± 0.7 Ba	56.6 ± 3.1 BCbc	46.6 ± 0.8 BCb	46.7 ± 0.6 Cb	702.9 ± 2.8 Cbc	140.3 ± 7.3 BCa	1.21 ± 0.05 Aa
	S4	10.6 ± 0.2 Cc	7.3 ± 0.6 Bbc	50.3 ± 2. 3 Cb	43.2 ± 0.6 Cb	44.4 ± 0.4 Cc	703.6 ± 2.4 Ca	126.2 ± 9.7 Ca	1.16 ± 0.04 Aa
T3	S1	14.2 ± 0.4 Aa	10.4 ± 0.4 Aa	82.2 ± 2.6 Aa	62.8 ± 2.2 Aa	76.1 ± 2.7 Aa	918.3 ± 5.1 Aa	170.9 ± 3.3 Aa	1.31 ± 0.01 Aa
	S2	12.6 ± 0.3 Bc	8.3 ± 0.5 Ba	60.1 ± 3.4 Bb	46.2 ± 0.8 Bb	56.2 ± 1.3 Bb	821.4 ± 4.2 Bc	145.5 ± 1.8 Ba	1.30 ± 0.05 Aa
	S3	10.2 ± 0.3 Cc	7.4 ± 0.6 Ba	51.2 ± 3.2 BCc	44.1 ± 0.7 Bb	43.2 ± 0.3 Cc	691.5 ± 2.9 Cc	134.3 ± 2.16 Ba	1.16 ± 0.06 Aa
	S4	9.3 ± 0.1 Cd	7.1 ± 0.3 Bc	46.3 ± 1.6 Cb	38.6 ± 0.6 Cc	40.6 ± 0.3 Cd	687.6 ± 1.8 Cb	120.2 ± 6.87 Ca	1.20 ± 0.03 Aa

Data are presented as the means ± standard error (*n* = 3). Different uppercase letters indicate significant differences among sampling periods, while different lowercase letters indicate significant differences among drought treatments within the same period (*p* < 0.05, according to Duncan’s test for one-way ANOVA).

## Data Availability

The raw data supporting the conclusions of this article will be made available by the authors upon request. The data are not publicly available due to the fact that they are part of an ongoing research project.
